# Cloning and molecular characterization of thiol-specific antioxidant gene of Leishmania tropica Turkey isolate

**DOI:** 10.3906/sag-1808-98

**Published:** 2019-02-11

**Authors:** Hamza ÖZAVCI, Mustafa KAPLAN

**Affiliations:** 1 Department of Medical Parasitology, Faculty of Medicine, Fırat University, Elazığ Turkey

**Keywords:** Cutaneous leishmaniasis, Thiol-specific antioxidant, cloning, DNA vaccine

## Abstract

**Background/aim:**

Thiol-specific antioxidant (TSA) protein is one of the most promising molecules among candidates for vaccine against cutaneous leishmaniasis. It was found to be significantly protective against different *Leishmania* species. In this study, cloning and molecular characterization of thiol-specific antioxidant gene of *L. tropica* Turkey isolate (LtTSA) were aimed.

**Materials and methods:**

LtTSA was amplified by PCR using the specific primers of TSA gene and cloned into the pcDNA3.1 vector. The cloning was confirmed by PCR screening, restriction enzyme reactions, and DNA sequence analysis. Finally, three-dimensional structure and antigenic properties of the protein encoded by the LtTSA were determined.

**Results:**

Six hundred base pair bands belonging to LtTSA were shown with electrophoresis. It was found that LtTSA and its encoded protein have high similarity with different *Leishmania* species. LtTSA protein consisting of 199 amino acids was found to have 7 different antigenic regions.

**Conclusion:**

LtTSA and its encoded TSA protein were found to be highly immunogenic and similar to TSA proteins previously tested as a vaccine candidate.

## 1. Introduction

Leishmaniasis is a zoonotic disease caused by protozoan *Leishmania* species. Leishmaniasis, one of the neglected tropical diseases, is prevalent especially in developing countries (1,2). According to the World Health Organization’s current estimates, approximately 12 million people in 98 countries are infected with *Leishmania* species and 350 million people are at risk (3). Cutaneous leishmaniasis (CL), the most common clinical form of the disease, is seen in 0.7–1.3 million people per year (4). It was reported that it is spreading rapidly all over the world due to difficulties in fighting vectors, travel, and migration, wars, and increase in the number of individuals with immunodeficiency (5–7). 

CL usually heals spontaneously, but scar tissue leaving a permanent mark at the infection site causes cosmetic problems in patients (1,8). In addition, factors such as the cost of drugs used in the treatment of infection, their toxic effects, and drug resistance have increased the importance of vaccination studies against CL (9,10). 

In vaccination studies, dead and attenuated parasites with reduced pathogenicity were used initially, but adequate protection was not achieved (9). Then, *Leishmania* antigens such as lipophosphoglycan (LPG), glycoprotein 63 (GP 63) proteins, fucose mannose ligand (FML) protein (11), and recombinant antigens such as *Leishmania* homologue of receptor for activated C kinase (LACK), cysteine protease type-I (CPB), cysteine protease type-II (CPA), cysteine protease type-III (CPC), glucose-regulated protein 78 (GRP78), promastigote surface antigen-2 (PSA-2), hydrophilic acylated surface protein B1 (HASPB1), kinetoplastid membrane protein (KMP-11), *Leishmania* *major* stress-inducible protein-1 (LmSIP1), *Leishmania* elongation initiation factor (LeIF), and thiol-specific antioxidant (TSA) were tested as vaccine candidates (12–21). In all the vaccine candidates developed using these, partial protection against different *Leishmania* species was achieved but a protective commercial vaccine against the disease has not been produced yet. However, immunization studies with Leish-111f and Leish-F2 proteins, the most promising vaccine candidate molecules, have progressed to phase-II (15). 

One of the most widely used molecules in vaccination studies against CL is TSA protein. So far, it has been tested as a vaccine candidate against many different *Leishmania* species (10,12–14,21–23). This protein, homologous to the eukaryotic TSA protein, is secreted in both amastigote and promastigote forms in *Leishmania* species. 15. Consisting of 200 amino acids and weighing 22.1 kDa molecules, it is encoded by the TSA gene localized on the chromosome (16,18). It has been reported that the TSA protein is strongly antigenic for both mice and humans and induces a Th1-dependent immune response in the host body, particularly by strongly stimulating cellular immunity. In addition to causing IgG2a production, specific IgG1 has been shown to cause a high level of IFN-γ and low level of IL-4 release (13,14,21).

In recent years, DNA vaccines, which are superior to protein vaccines, have been used since they are more stable, cheaper to produce, able to host more than one gene, and they do not need cold chain during transport (24–28). In this context, different levels of protection against infection in experimental animals have been achieved with DNA vaccines containing genes encoding glycoprotein 63 (Gp 63), glycoprotein 46 (Gp 46), PSA-2, CpA + CpB, KMP-11, NH, NH-36, LPG 3, open reading frame fragment (ORFF), amastigote antigens (A2, P4, P8), histone proteins (H2A, H2B, H3, H4), *L. infantum* acidic ribosomal protein P0 (LiPO), proteophosphoglycan (papLe22), LACK, LeIF, TSA and LmSIP-1 proteins (11, 28). 

Despite all immunization studies against CL with different molecules, a fully effective and protective vaccine has not been produced yet. In addition, research is usually on *L. major*, which is the most common CL causative agent in the world. There are also studies on different CL causative agents such as *L. aethiopica* and *L. braziliensis* (11,17,20,24). However, the number of research related to *L. tropica*, which is the most frequent causative agent in Turkey, is fairly limited (29). 

Experimental studies using TSA-containing DNA and recombinant protein vaccines for different *Leishmania* species have been found to provide promising protection against CL (11,12,14,17,20,21,23). Although it is the most common species in Turkey, DNA vaccine studies against *L. tropica* are insufficient. In this study, in the scope of the fight against CL in our country to develop a DNA vaccine against *L. tropica*, molecular characterization of the TSA gene belonging to the *L. tropica* Turkey isolate and its cloning into pcDNA3.1 vector was aimed.

## 2. Materials and methods

### 2.1. L. tropica promastigote culture 

*L. tropica* promastigotes isolated from a patient suffering from CL, who applied to the Medical Parasitology Department of Erciyes University Medical Faculty, were propagated in 15% fetal bovine serum (FBS) (Biochrom, Berlin, Germany), 100 μg/mL streptomycin-100 IU/mL penicillin (Invitrogen Corp., Waltham, MA, USA), and RPMI-1640 (Biochrom) medium containing 1 mL of L-glutamine (Invitrogen) (20). 

## 2.2 Isolation, amplification, and purification of L. tropica TSA gene 

RNA was isolated from promastigotes found in the culture at stationary phase using the TRI Reagent T3809 RNA isolation kit (Becton Dickinson, USA). The acquired RNA was used as a template and the cDNA was obtained from *L. tropica* RNA by working in accordance with the procedure of Easyscript Plus cDNA Synthesis Kit (ABM, Canada). LtTSA was then amplified using LtTSA gene specific F [LtTSA F: (5’-GCCATGTCCTGCGGTGAAACCAA-3’)] and R [LtTSA R: (5’- TTACTGTTTGCTGAAGTACCCCT- 3’)] primers designed with reference to the sequence of DQ071683. One numbered sample was found in the GenBank database. For this, a total of 25 μL of PCR mixture consisting of 12.5 μL of Master Mix (Biomatik, USA), 9.5 μL of distilled water, 1 μL of cDNA, and 1 μL of LtTSA F (20 pmol) and 1 μL of LtTSA R (20 pmol). Then a thermal cycler (Sensoquest) was operated with a cycle of 5 min at 95 °C; 1 min at 95 °C; 1 min at 53 °C; 1 min 35 cycles at 72 °C; 10 min at 72 °C, and the mixture was rested at +4 °C in the last step. The obtained PCR product was moved through a 1.5% agarose gel (Lonza) for 45 min at 120 V and the resulting bands were viewed with a ChemiDoc Mp Gel Imaging System (Bio-Rad, Berkeley, CA, USA). LtTSA was purified from the gel using the Wizard®SV Gel & PCR Clean-Up System (Promega, Fitchburg, WI, USA) kit.

### 2.3. Cloning Of LtTSA gene

#### 2.3.1. Ligation and transformation 

The purified LtTSA gene was cloned using pcDNA™3.1/V5-His TOPO® TA Expression Kit (Invitrogen) and the recombinant plasmid containing LtTSA gene was obtained as a result of ligation. The recombinant plasmid was transferred into One Shot ® TOP10 chemically competent *E. coli* (Invitrogen) cells and the competent *E. coli* were then taken to 250 μL of S.O.C. Medium (ThermoFisher Scientific, Waltham, MA, USA) fluid broth medium. After being incubated at 37 °C in a shaking incubator for 90 min at 220 rpm, the cells were seeded on an LB agar plate containing Ampicillin and grown at 37 °C overnight. 

#### 2.3.2. PCR screening

The presence of recombinant plasmids in the competent *E. coli* colonies which multiplies in the LB agar was controlled by PCR screening. For this procedure, 25 μL of PCR mixture consisting of 12.5 μL of 2X Master Mix (Biomatik), 1 μL of LtTSA F primer, 1 μL of LtTSA R primer, and 10.5 μL of distilled water was prepared and 0.5 mL of randomly selected colonies was added onto it. It was then amplified by application of a cycle of 5 min at 95 °C; 1 min at 95 °C; 1 min at 53 °C; 1 min 35 cycles at 72 °C; 10 min at 72 °C and resting at +4 ˚C in the last step. The obtained PCR product was moved through a 1% agarose gel (Lonza, Basel, Switzerland) with Ethidium Bromide for 45 min at 120 V and the resulting bands were viewed with a ChemiDoc Mp Gel Imaging System (Bio-Rad).

#### 2.3.3. Miniprep (purification)

Recombinant plasmid-bearing *E. coli* colonies were incubated in liquid LB broth containing Ampicillin and shaken overnight at 37 °C. Using the GeneAll Exprep Plasmid SV Mini kit (GeneAll Biotechnology), miniprep was performed on the grown *E. coli* cells and recombinant plasmids were purified. Whether these plasmids contained the LtTSA gene was tested by PCR, DNA sequence analysis, and restriction enzyme reactions. 

### 2.4. DNA sequence analysis

Three different DNA sequence analyses of the resulting recombinant plasmid were performed using the F primer of LtTSA F, LtTSA R, and pcDNA3.1/ V5-his TOPO® plasmid. Sequence results were compared with each other and the resulting LtTSA gene sequencing was finalized. Subsequently, the obtained gene sequence was compared with the TSA gene sequences of other *Leishmania* species in the literature and the similarities and differences were revealed. 

### 2.5. Determination of TSA protein structure

The sequence of the LtTSA gene was entered into the Emboss Transeq 6.6.0 program and the amino acid sequence coded by this gene was determined and the amino acid differences with the TSA proteins belonging to other *Leishmania* species have been identified. The three-dimensional structure of the protein encoded by the LtTSA gene was then created using Protein Homology Analogy Recognition Engine V 2.0 (PHYRE 2) program. Finally, the antigenic index of this protein was determined by the Kolaskar and Tongaonkar method (30).

### 2.6. Restriction enzyme reactions

In order to confirm the cloning, recombinant plasmid was cut using HindIII with NotI enzymes and HindIII with XhoI enzymes (New England Biolabs, UK). For this purpose, two different restriction reaction mixtures were prepared. 10 μL (1 mg) of the recombinant LtTSA plasmid product was added to the first tube containing 2 μL of HindIII with NotI enzyme, 5 μL 10X NE Buffer (New England Biolabs, UK), and 31 μL of distilled water and to the second tube containing 2 μL of HindIII with XhoI enzyme, 5 μL of 10X NE Buffer, and 31 μL of distilled water. After the prepared mixtures were incubated overnight at 70 rpm in a hot water bath at 37 °C, the enzymatic activity was terminated by adding 10 μL of 6X Loading Buffer (New England Biolabs, UK) solution to both tubes. Then, 25 μL of the contents of both tubes were removed and were let move through a 1% agarose gel (Lonza) and cloning was confirmed by visualization.

## 3. Results

### 3.1. L. tropica culture, LtTSA gene isolation, amplification, and purification 

**Figure 1:**
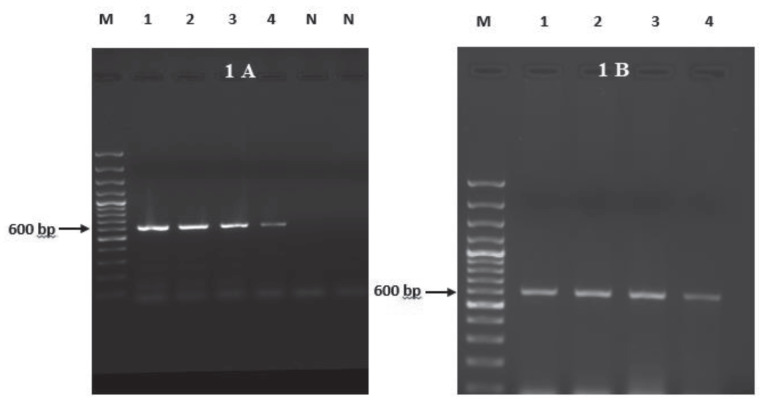
A: Amplification of LtTSA gene by PCR, B: Clean-up product. (M: Marker (100 bp), 1–4: TSA (+) PCR products, N: Negative control)

The concentration of RNA isolated from *L. tropica* promastigotes cultured in vitro on RPMI-1640 medium containing 15% FBS was measured as 1.206 ng/μL. This RNA was then used as a template to obtain cDNA at a concentration of 146.3 ng/μL. The PCR reaction, designed using TSA gene specific primers, resulted in bands at a size of 600 bp (Figure 1A). The amplified LtTSA gene was purified from the agarose gel using the Wizard®SV Gel & PCR Clean-Up System (Promega) kit. The obtained product was visualized with ChemiDoc Mp Gel Imaging (BioRad) and bands of 600 bp size belonging to LtTSA gene were observed (Figure 1B).

### 3.2. Cloning of LtTSA gene and PCR screening

**Figure 2:**
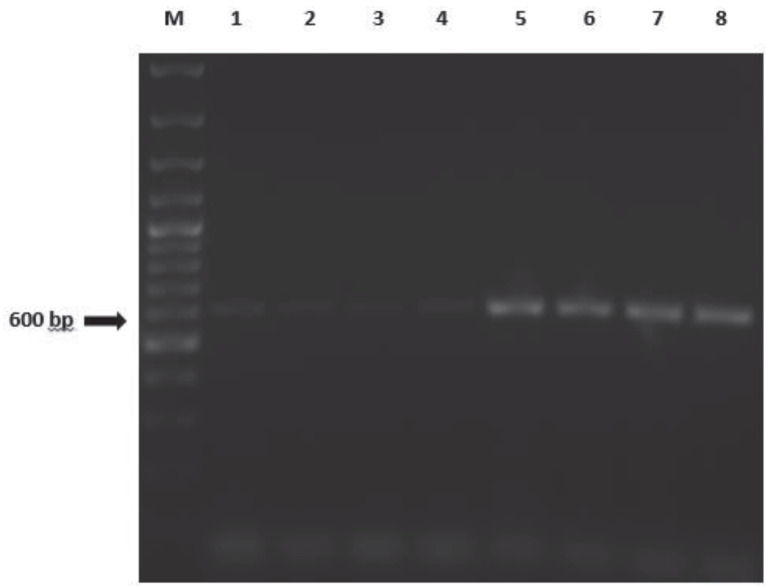
PCR screening result (M: Marker (100 bp), 1–8: (+) Colonies)

After the purified LtTSA gene was transferred to pcDNA3.1 / V5-his TOPO plasmid, it was transformed into competent *E. coli* cells and cultured on solid LB medium containing ampicillin. The presence of recombinant plasmids in the resulting colonies was demonstrated by PCR screening using specific primers (Figure 2).

### 3.3. Purification of recombinant plasmid

**Figure 3:**
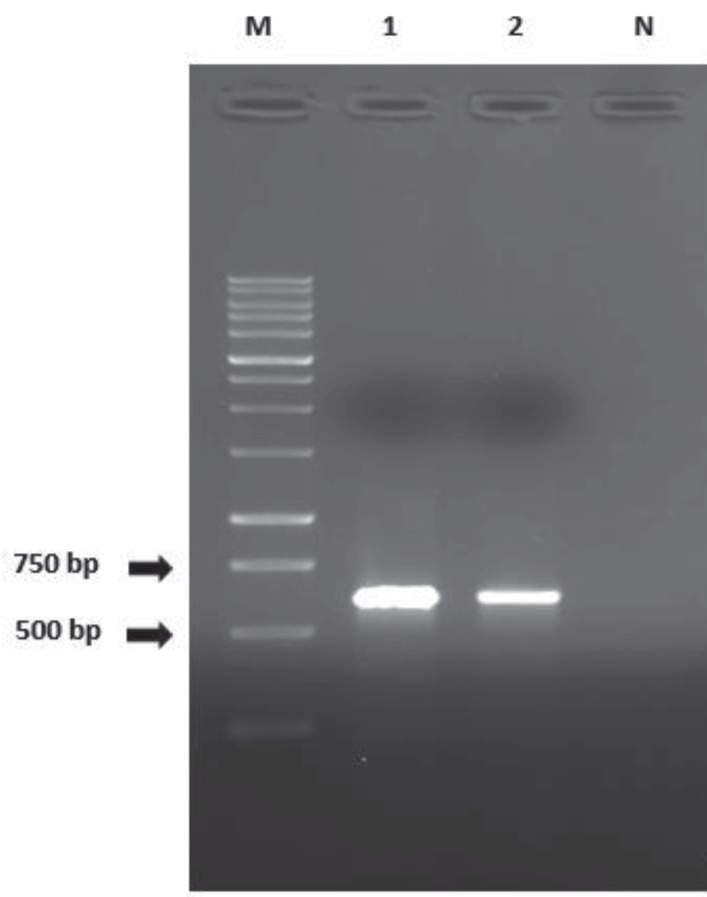
Demonstration of LtTSA genes in recombinant plasmids. (M: Marker (1 kb), 1–2: Miniprep product, N: Negative control)

Miniprep was performed to purify recombinant plasmids containing the LtTSA gene from competent *E. coli* cells. The presence of LtTSA gene was demonstrated in recombinant plasmids by PCR reaction designed using specific primers (Figure 3). 

### 3.4. DNA sequence analysis

Cloning of the LtTSA gene was validated by sequence analysis and restriction enzyme reactions and recorded in GenBank database with KX259106 number (Table 1).

**Table 1:** LtTSA gene nucleotide sequence.

1	ATG	TCC	TGC	GGT	GAA	ACC	AAG	ATC	AAC	TCT	CCC	GCG	CCG	CCC	TTC
46	GAG	GAG	ATG	GCG	CTC	ATG	CCC	AAC	GGC	AGC	TTC	AAG	AAG	ATC	AGC
91	CTC	TCC	GCC	TAC	AAG	GGC	AAG	TGG	GTC	GTG	CTC	TTC	TTC	TAC	CCG
136	CTC	GAC	TTC	ACC	TTC	GTG	TGC	CCG	ACA	GAG	ATC	ATC	GCG	TTC	TCC
181	GAC	AAC	GTG	AGT	CGC	TTC	AAC	GAG	CTC	AAC	TGC	GAG	GTC	CTC	GCG
226	TGC	TCG	ATG	GAC	AGC	GAG	TAC	GCG	CAC	CTG	CAG	TGG	ACG	CTG	CAG
271	GAC	CGC	AAG	AAG	GGC	GGC	CTC	GGG	GCC	ATG	GCG	ATC	CCA	ATG	CTG
316	GCC	GAC	AAG	ACC	AAG	TGC	ATC	GCT	CGT	TCC	TAC	GGC	GTG	CTG	GAG
361	GAG	AGC	CAG	GGC	GTG	GCC	TAC	CGC	GGT	CTC	TTC	ATC	ATC	GAC	CCC
406	CAT	GGC	ATG	GTG	CGT	CAG	ATC	ACC	GTC	AAC	GAC	ATG	CCG	GTG	GGC
451	CGC	AAC	GTG	GAG	GAG	GTT	CTG	CGC	CTG	CTG	GAG	GCT	TTT	CAG	TTC
496	GTG	GAG	AAG	CAC	GGC	GAG	GTG	TGC	CCC	GCG	AAC	TGG	AAG	AAG	GGC
541	GCC	CCC	TCG	ATG	AAG	CCG	GAA	CCG	AAG	GCG	TCT	GTC	GAG	GGG	TAC
586	TTC	AGC	AAA	CAG	TAA*	600									

* Stop codon.

**Figure 4:**
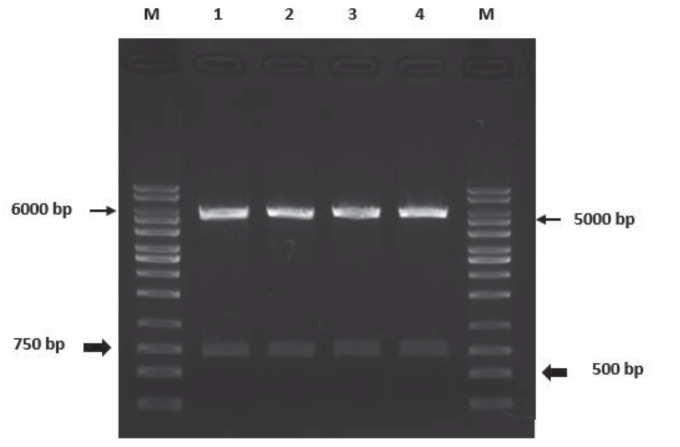
Result of cutting recombinant plasmids with restriction enzymes. (M: Marker (1 kb), 1–4: Restriction products)

Two different size bands were observed in the 500–750 bp and 5000–6000 bp range as a result of the restriction process using HindIII with XhoI and HindIII with NotI enzymes. From these, the bands in the range of 500–750 bp were the plasmid fragment containing the LtTSA gene with 600 bp size and the bands in the range of 5000–6000 bp were found to be bands showing the rest of the recombinant pcDNA3.1 / V5-his TOPO plasmid in 6123 bp size after the restriction process (Figure 4).

The nucleotide sequence of the LtTSA gene was compared with the nucleotide sequences of the TSA genes of the *Leishmania* species registered in the GenBank database. It was found that LtTSA gene showed 99% similarity with Ethiopia isolate of *L. tropica* (Table 2). In addition, it was also found to be highly similar to TSA gene sequences belonging to different *Leishmania* species, such as *L. aethiopica* (99%), *L. donovani* (97%), *L. infantum* (97%), *L. chagasi* (97%), *L. major* (97%), and *L. mexicana* (93%) (Table 3).

**Table 2:** Comparison of LtTSA gene nucleotide sequence with L. tropica TSA genes in other countries.

GenBank no.Country	Different nucleotide regions		
	31.	489.	547.
KX259106 *Turkey	T	T
T	DQ071683.1Ethiopia	A	G	A

* Strain defined in this study.

**Table 3:** Comparison of LtTSA gene nucleotide sequence with TSA gene of other Leishmania species.

LeishmaniaspeciesGenBank no.	Country	13.	15.	16.	21.	24.	28.	29.	30.	31.	33.	40.	51.	52.	94.	110.	136.	137.	138.	146.	171.	172.	173.	183.	185.	190.	191.	216.	220.	231.	233.	277.	294.	295.	306.	331.	332.	343.	344.	359.	364.	365.	366.	367.	406.	412.	414.	415.	420.	455.	525.	542.	547.	549.	558.	561.	570.	574.	576.	579.	594.	Different nucleotideregions
L.tropica* KX259106	Turkey	G	A	A	G	C	T	C	T	C	C	C	G	A	T	A	C	T	C	C	C	G	C	C	A	A	G	G	C	G	T	A	G	G	C	T	G	T	C	A	A	G	C	C	C	A	G	G	T	A	G	C	T	G	G	A	G	G	C	G	A	**
L.aethiopicaDQ071684.1	Ethiopia		C	G										G											G							C																		G			A									99%
L.donovaniFR799602.2	Nepal		C	G										G	G									A						C			C		T	A		G				A	A		A								A			G						97%
L. infantumFR796447.1	Spain		C	G				G						G	G									A						C			C		T	A		G		C		A	A		A								A			G						97%
L. chagasi AF312397.1	Brazil		C	G				G						G	G															C			C		T	A		G		C		A	A		A				C				A			G						97%
L. majorEU194915.1	Iran	A	C	G				G						G	G									A						C			C		T	A		G				A	A		A								A			G					G	97%
L. mexicanaFR799568.1	Guatemala		C	G				G	C	G		G				G	T	A	T	G	T	C	A			G	C	C	A	C	C		C	A			C	G	T	C	-			A		G	C	C	C		C	A	A	A	A		A	A	T	A		93%

* Strain defined in this study, **Similarity rate.

It was determined that the LtTSA gene consists of 600 nucleotides and codes for the TSA protein with 199 amino acids. The amino acid sequence of this protein was determined using the Emboss Transeq 6.6.0 program (Table 4).

**Table 4:** Amino acid sequence of LtTSA protein.

Met	S	C	G	E	T	K	I	N	S	P	A	P	F	E	E	Met	A	L	Met
P	N	G	S	F	K	K	I	S	L	S	S	A	Y	K	G	K	W	V	V
F	F	Y	P	L	F	T	F	V	C	P	E	I	I	A	F	S	D	N	V
S	R	F	N	E	L	C	E	V	L	A	C	S	Met	D	S	E	Y	A	L
H	Q	W	T	L	Q	D	R	K	K	G	G	L	G	A	Met	A	I	P	Met
L	A	D	K	T	K	C	I	A	R	S	Y	G	V	L	E	E	S	Q	G
V	A	Y	R	G	L	F	I	I	D	P	H	G	Met	V	R	Q	I	T	V
N	D	Met	P	V	G	R	N	V	E	E	V	L	R	L	L	E	A	F	Q
FV	E	K	H	G	E	V	C	P	A	N	W	K	K	G	A	P	S	Met	K
P	E	P	K	A	S	V	E	G	Y	F	S	K	Q	*					

*Stop kodon.

Similarities and differences were shown by comparing the amino acid sequence of the TSA proteins of the *Leishmania* species registered in the GenBank database with the LtTSA protein (Table 5). The TSA protein encoded by the LtTSA gene was found to be 98% similar to the TSA protein and 96%, 94%, and 93% to *L. aethiopica*, *L. infantum* and *L. major* TSA proteins, respectively.

**Table 5:** Comparison of LtTSA protein with TSA proteins of other Leishmania species.

GenBank no.Leishmania speciesCountry	5.	6.	10.	18.	32.	33.	56.	61.	62.	67.	78.	93.	99.	102.	104.	111.	115.	122.	136.	139.	152.	163.	179.	183.	Different aminoacidregions
KX259106 *L. tropicaTurkey	E	T	S	M	S	A	I	D	N	N	M	K	A	I	M	C	S	S	H	V	N	F	K	S	**
AAZ23601.1L. tropicaEthiopia																S						L		T	98%
AAZ23602.1L. aethiopicaEthiopia	D	A		V					S			Q									S			T	96%
AAK58478.1L. infantumSpain			C	V	A			E						T		S	A	K	N				N	T	94%
AGN89094.1L. majorPakistan						S	V		S	S	I		T		I	N				L	S				93%

* Strain defined in this study, ** Similarity rate.

**Figure 5:**
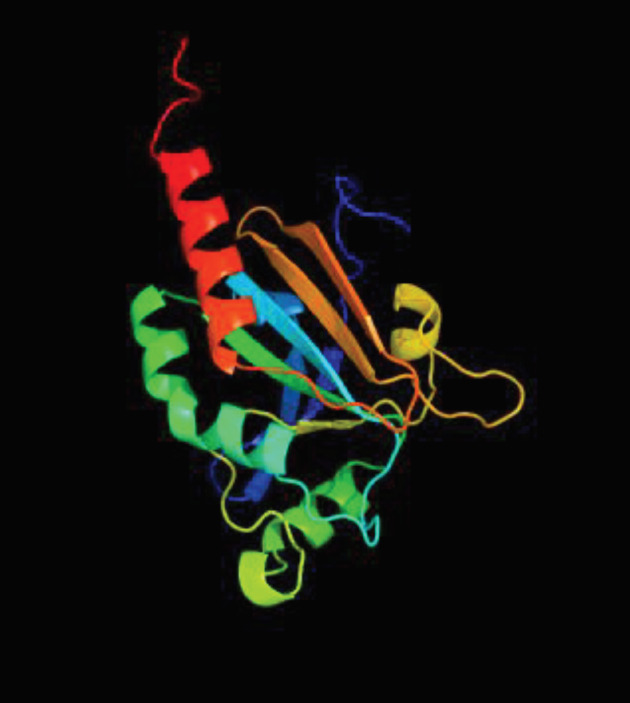
Three dimensional structure of LtTSA protein.

The three-dimensional structure of the LtTSA protein was created with “Protein Homology Analogy Recognition Engine Version 2.0 (PHYRE 2) program (Figure 5).

**Figure 6:**
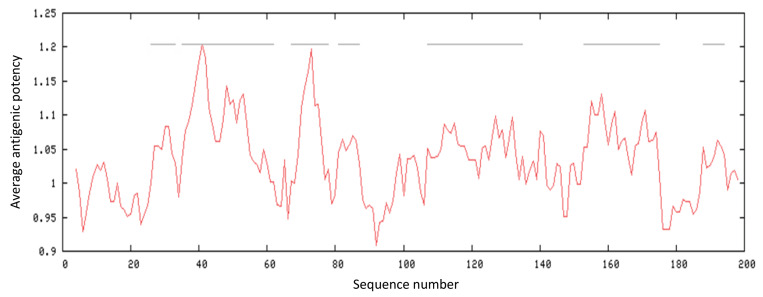
Antigenic index of LtTSA protein.

Finally, the antigenic index of LtTSA protein was calculated with the Kolaskar and Tongaonkar method and found as 1.0321 (Figure 6). 

The LtTSA protein, consisting of 199 amino acids, was found to have a total of 7 different antigenic regions and the amino acid sequences of the antigenic regions, the start and end positions of the LtTSA protein were shown in Table 6. Among these antigenic determinants, regions 2 and 3 were found to be the most immunogenic regions.

**Table 6:** Antigenic regions of LtTSA protein.

n	Initial position	Aminoacid sequence	End position
1	26	FKKISLSA	33
2	35	KGKWVVLFFYPLDFTFVCPTEIIAFSDN	62
3	67	NELNCEVLACSM	78
4	81	EYAHLQW	87
5	107	DKTKCIARSYGVLEESQGVAYRGLFIIDP	135
6	153	VEEVLRLLEAFQFVEKHGEVCPA	175
7	188	PKASVEG	194

n: Antigenic region number.

## 4. Discussion

The average number of CL cases, which is 0.7–1.3 million per year, continues to increase rapidly in our country and all over the world due to the difficulties and inadequacies in the fight against the disease (4,5,7). This increases the importance of producing a protective vaccine against the disease, which is a serious public health problem (3,6,31–35). 

Different vaccine types have been tried against CL so far. The process that started with dead parasite vaccines continued with attenuated live vaccines, recombinant antigen vaccines, and DNA vaccines, and studies have been carried out with many different antigens (3,10–12,14–16,19–21,24,25,36–41). Different antigens such as LPG, GP 63, FML, LACK, cysteine proteases, GRP78, PSA-2, HASPB1, KMP-11, LmSIP1, LeIF, and TSA have been tested as vaccine candidates among *Leishmania* proteins (11–21). One of the molecules with the most promising results in vaccination studies against CL among these vaccine candidates is the TSA protein due to its superior properties, such as being able to be secreted by all forms of *Leishmania* species, stimulation of both humoral and cellular immunity, inducing strong Th1 immune response (11,15,24–27,41–44).

TSA protein has been used as a standalone vaccine candidate as well as a component of multi-antigenic vaccine candidates. It has been found to be more successful in multi-antigenic vaccine studies than when it was tried alone (12,14,45,46). It has been found that the vaccines containing a combination of TSA protein with *Leishmania* homologue of receptor for activated C kinase (LACK) protein (14) and *L. major* stress inducible protein 1 (LmSIP1) protein (12) and vaccines including TSA protein and LEISH-F2 (15,47) and Leish-111f proteins (12,19,36,39,46) combination can provide a highly effective protection against *L. major* infection. Among these, the multi-antigenic vaccine candidates LEISH-F2 and Leish-111f could reach Phase 2 in clinical trials (15). 

Although mice immunized with *L. braziliensis* TSA antigen did not develop any protective immunological response against *L. braziliensis* after infection and there was no significant reduction in lesions. This was interpreted as being specific to *L. braziliensis* (20).

In this study, it has been revealed that the LtTSA protein obtained from the isolate of *L. tropica* in Turkey has a very high antigenic index and it has a high similarity to TSA proteins of other *Leishmania* species. Given these characteristics, we believe that LtTSA protein will be a very important component for vaccines to be produced against *L. tropica* and that it will be as successful as the results obtained with other TSA vaccines. 

DNA vaccines are obtained by placing genes that encode the target protein into the mammalian expression vector. DNA vaccines are more advantageous than protein vaccines because they can contain more than one gene, have a more stable structure, do not need a cold chain during transport and their production cost is lower (24–28).

Different types of plasmids which are used for different results in recombinant DNA technology exist today, such as pBR322, pUC18, pUMVC, pCDNA.3.1, pVax1, pSMART, pDNAVACC, pCI-neo, pTARGET, and pFLAG (48). The vector pCDNA.3.1 used in this study is preferred due to its advantages such as high efficiency expression in a short time and in a single step, without requiring any ligase reaction for the cloning reaction and a post-PCR procedure. In addition, the pcDNA. 3.1 vectoris was preferred because it can multiply in mammalian cells, so it can create a starting point for DNA vaccine studies (49). 

The species that cause clinical infections and are frequently seen in dogs, the most important reservoir of leishmaniasis, are *L. infantum* and *L. tropica* (50). We believe that the LtTSA-based DNA vaccine candidate we obtained in our study can be used in the immunization of reservoir dogs as well as being used in humans for the prevention of CL and it could be a useful tool in combating leishmaniasis.

## Acknowledgments

We thank Prof. Dr. Salih Kuk and Prof. Dr. Süleyman Yazar for their support. Also, we wish to acknowledge the staff of Erciyes University Faculty of Medicine for their technical assistance.
